# Non-linear optical microscopy of cartilage canals in the distal femur of young pigs may reveal the cause of articular osteochondrosis

**DOI:** 10.1186/s12917-017-1197-y

**Published:** 2017-08-22

**Authors:** Andreas Finnøy, Kristin Olstad, Magnus B. Lilledahl

**Affiliations:** 1 Department of Physics, Norwegian University of Science and Technology (NTNU), Trondheim, Norway; 2 Faculty of Veterinary Medicine and Biosciences, Equine Section, Norwegian University of Life Sciences, P.O. Box 8146, Oslo, Norway

**Keywords:** Osteochondrosis, Cartilage canals, Chondronecrosis, Chondrification, Collagen, Shg, Tpef

## Abstract

**Background:**

Articular osteochondrosis is a common cause of leg weakness in pigs and is defined as a focal delay in the endochondral ossification of the epiphysis. The first demonstrated steps in the pathogenesis consist of loss of blood supply and subsequent chondronecrosis in the epiphyseal growth cartilage. Blood vessels in cartilage are located in cartilage canals and become incorporated into the secondary ossification centre during growth. It has been hypothesized that vascular failure occurs during this incorporation process, but it is not known what predisposes a canal to fail. To obtain new information that may reveal the cause of vascular failure, the distal femur of 4 pigs aged 82–140 days was sampled and examined by non-linear optical microscopy. This novel technique was used for its ability to reveal information about collagen by second harmonic generation and cellular morphology by two-photon-excited fluorescence in thick sections without staining. The aims were to identify morphological variations between cartilage canal segments and to examine if failed cartilage canals could be followed back to the location where the blood supply ceased.

**Results:**

The cartilage canals were shown to vary in their content of collagen fibres (112/412 segments), and the second harmonic and fluorescence signals indicated a variation in the bundling of collagen fibrils (245/412 segments) and in the calcification (30/412 segments) of the adjacent cartilage matrix. Failed cartilage canals associated with chondronecrosis were shown to enter the epiphyseal growth cartilage from not only the secondary ossification centre, but also the attachment site of the caudal cruciate ligament.

**Conclusion:**

The variations between cartilage canal segments could potentially explain why the blood supply fails at the osteochondral junction in only a subset of the canals. Proteins linked to these variations should be examined in future genomic studies. Although incorporation can still be a major cause, it could not account for all cases of vascular failure. The role of the caudal cruciate ligament in the cause of osteochondrosis should therefore be investigated further.

## Background

Articular osteochondrosis is a developmental orthopaedic disease defined as a multi-focal disturbance of the endochondral ossification of the epiphysis [1] and is a major cause of leg weakness in pigs [2, 3] and lameness in other species [4]. The advanced stage of the disease is characterized by loosening of osteochondral fragments that can expose the subchondral bone, a condition known as osteochondritis dissecans [5]. The incidence of osteochondrosis in certain breeds of domesticated pigs is estimated to be above 80% [6, 7]. Several factors have been suggested to contribute to the disease development, including genetic factors [1], joint biomechanics [8], and inhomogeneous mechanical properties of the subchondral bone [9] and the epiphyseal growth cartilage [10, 11]. However, the estimates of heritability vary substantially, in the range of 0.06 to 0.49 [12, 13], and the disease development is likely governed by different factors at different stages [1, 14]. Studies of early lesions showed that the focal delay in ossification was caused by areas of chondronecrosis in the sub-articular epiphyseal growth cartilage that resisted resorption and replacement by bone [15–17]. These findings were corroborated by experimental studies where the blood supply to limited areas of the distal femur was transected in pigs [18, 19], horses [20], and goats [21] and produced lesions similar to spontaneous osteochondrosis. To date, the first step in the pathogenesis is therefore considered to be focal failure in the blood supply to the epiphyseal growth cartilage [14]. However, the cause of the vascular failure remains to be clarified.

**Fig. 1:**
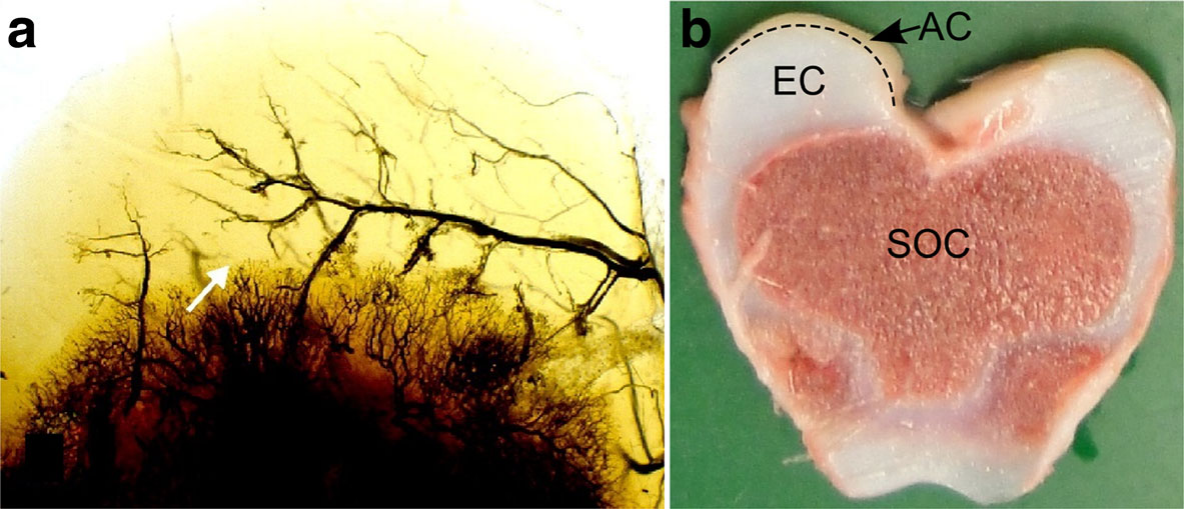
Epiphyseal growth cartilage. **a** Blood vessels in the medial femoral condyle of an 11-week old pig. The sample has been cleared in 100% methylsalicylate, and the blood vessels were perfused with barium sulfate to enhance contrast. An arterial blood vessel penetrating the epiphyseal growth cartilage from the perichondrial plexus is called a vascular trunk. In the image, a vascular trunk is running parallel to the ossification front with perpendicular-oriented branches. The arrow points to a necrotic cartilage canal that was observed during histological examination. Included with permission from [17]. **b** 5 mm thick slab from the distal femur of a 20 kg pig. A cross-section of the secondary ossification center (SOC) and the epiphyseal growth cartilage (EC) can be observed. The approximate transition to the immature articular cartilage (AC) is indicated by a dashed line on the medial femoral condyle

Blood vessels in epiphyseal growth cartilage are temporarily present and run through tubular passages known as cartilage canals [22]. Each cartilage canal contains a capillary bed that is supplied by a single arteriole originating from the perichondrial plexus and drained by one or more venules [23]. The cartilage canals form vascular trees with regularly spaced and blind-ending branches [24] (see Fig. 1a). In addition to the nutritive role, the cartilage canals have perivascular mesenchymal cells that can contribute to chondrogenesis [25] and osteogenesis in the secondary ossification centre (SOC) [26]. During skeletal growth, the epiphyseal growth cartilage acts as a scaffold for the SOC and is gradually replaced by bone until only the layer of mature articular cartilage remains [27]. The cartilage canals are normally incorporated into the advancing ossification front [22]. Simultaneously, the distal terminus of some cartilage canal branches are converted into cartilage in a physiological process referred to as chondrification [22, 28]. This is a poorly understood process that involves loss of endothelial cells and differentiation of perivascular mesenchymal cells into chondrocytes [18, 17], but chondrification occurs in a predictable pattern and is not associated with ostochondrosis [22].

To determine the cause of vascular failure, animals should be examined in the age window when the cartilage canals are present [29]. The prevailing hypothesis states that the blood supply fails when the cartilage canals are being incorporated into the SOC and need to cross the osteochondral junction [17, 30]. To test this, the cartilage canals should be examined prior to or at the time of vascular failure before the advancing SOC removes traces of structural changes from the incident. Furthermore, the blood supply can fail in a single canal and leave most of the neighbouring canals intact [31]. A better understanding of the variation between cartilage canals prior to failure is therefore necessary. In young horses, collagen type I fibres were observed around a subset of cartilage canals in histological sections [32]. Collagen type I fibres and collagen type II fibrils have different mechanical properties [33] and may provide different protection during the incorporation process. We hypothesize that a similar variation in collagen is present around cartilage canals of young pigs.

In this preliminary study, we examined the possibility of using non-linear optical microscopy to obtain information about cartilage canals and the cause of vascular failure in young pigs. Non-linear optical microscopy was first applied to cartilage in 2005 [34] and is based on a laser that emits high-intensity light in short pulses. The light induces a non-linear optical response, limited to a small volume around the focus, so that more than 100 μm-thick sections can be imaged without using a pinhole. In contrast to conventional histological techniques, 3D information about morphological changes can be obtained with less labour-intensive and artefact-prone serial sectioning [35]. Although large field of view in 3D can be provided by MRI and CT, these methods have limitations in resolving morphological changes at microscopic scale [31, 36]. An effect of the non-linear response is excitation of endogenous fluorophores by two photons simultaneously, known as two-photon-excited fluorescence (TPEF). The TPEF signal was previously used to visualize cells in the cartilage of young animals [37, 38]. This is possible when the autofluorescence from the cross-links in the collagen matrix is lower than from the fluorophores of the cell [39]. Collagen fibrils mediate another non-linear response through frequency-doubling of light, known as second-harmonic generation (SHG) [40]. The emitted SHG signal is highly specific to collagen fibrils [34], and when the imaging conditions are kept constant [38], the SHG intensity is sensitive to the density [41], thickness [42] and relative orientation of the collagen fibrils [43]. The cells and the collagen matrix can therefore be examined without staining.

The aims of this study were to use TPEF and SHG to i) identify morphological variations between segments of patent cartilage canals that may predispose to vascular failure and ii) examine if failed cartilage canals could be followed back to the location where the blood supply ceased.

## Methods

### Material

Four male Landrace pigs were included in the study. The animals originated from a previous study that was approved by the National Animal Research Authority [30]. The pigs were 82 days (33 kg), 96 days (47 kg), 127 days (67 kg) and 140 days (80 kg) old. Pigs in this age window are known to have patent cartilage canals, and the younger pigs have a higher number of cartilage canals than the older [22]. No age-dependent variation was examined in the current study, only variation between segments of cartilage canals. To increase the chance of developing osteochondrosis, the pigs were purpose-bred and sired by a single boar that had the highest risk of osteochondrosis among his offspring of all breeding boars used at the time in the region, as calculated based on macroscopic evaluation of slaughtered relatives [44].

### Sample preparation

The right femur of each pig was harvested and fixed in 4% phosphate-buffered formaldehyde for a minimum of 48 h. The samples were obtained from the medial and lateral femoral condyle. This was achieved by sawing the distal femur into approximately 5 mm thick slabs in a transverse plane, as shown in Fig. 1b. The slabs were cut into smaller blocks containing the medial or lateral femoral condyle. The blocks were then decalcified in 10% EDTA and sliced with a vibratome (Leica VT 1000s) into 100 μm thick sections perpendicular to the articular surface. The sections were mounted on microscope slides with 70% glycerol for examination with SHG and TPEF.

### Number of sections examined

To identify variable features around cartilage canal segments, 3 successive sections were examined from 2 blocks from each of the medial and lateral condyle of the 82-, 96- and 140-day old pigs. From the 127-day old pig, 3 successive sections from two different blocks of the lateral condyle were examined. In total 42 sections were observed using SHG and TPEF.

Tracking of cartilage canals without blood supply was achieved by first identifying a cartilage canal without intact blood vessels in one of the 42 sections and then following the failed canal through consecutive serial thick sections. The canal was followed until it crossed a boundary to the epiphyseal growth cartilage or was no longer available due to artefacts caused by slicing. The number of sections examined per canal ranged from one to 33.

### Conventional histology

A 1 mm section was cut with the vibratome adjacent to a 100 μm-section prepared for SHG and TPEF from the medial condyle of the 82 day-old pig. The 1 mm section was paraffin-embedded, sliced into 4 μm sections, and stained with hematoxylin and eosin (H&E) for histological examination and comparison with SHG and TPEF.

### Imaging with SHG and TPEF

The 100 μm-sections were imaged using a Leica TCS SP8 confocal microscope with a Chameleon Vision S (Coherent) mode locked Ti:Sapphire femtosecond laser. The samples were excited with the laser at 890 nm wavelength. A 445 ± 10 nm filter (Semrock) was used to detect SHG, while a 525 ± 25 nm filter (Semrock) was used for TPEF detection. A dichroic mirror at 490 nm was used to separate SHG from TPEF. The SHG produced in forward direction was detected using non-descanned detection [45]. The backward-directed SHG was not used in this study.

The laser was focused on the sample by different objectives to achieve different magnification. The focusing objectives were 10× (NA 0.4, mod nr. 11,506,285, Leica), 25× (NA 0.95, mod. nr. 11,506,374, Leica), and 63× (NA 1.2, mod. nr. 11,506,361, Leica). The transmitted light was collected by Leica S-28 NA 0.55, Leica S-1 NA 0.9, and Leica S-1 NA 1.4, respectively.

The integrated software (Leica Application Suite X) controlled the microscope and the laser. Appropriate contrast was achieved by adjusting the laser intensity. The mosaic merge function of the software was used to create images with large field of view.

The H&E-stained sections were imaged with a Nikon Diaphot microscope (Nikon) using a Nikon DS-Fi1 camera (Nikon).

#### Identification of cartilage canals using TPEF

The TPEF signal from erythrocytes, endothelial cells and perivascular cells was used to identify the cartilage canals. Cells were distinguished based on their morphology. The portion of the canal located furthest away from the arterial source was referred to as the distal terminus. Differentiation between patent, chondrifying and necrotic cartilage canals was done based on criteria used in conventional histology [18]. If intact endothelial cells were present, the canal was considered to be patent. Cartilage canals that lacked blood vessels with endothelial cells and contained chondrocytes were referred to as chondrifying. Necrotic cartilage canals were identified as canals without intact endothelial cells, presence of necrotic perivascular cells and necrotic chondrocytes adjacent to the canal. Cell shrinkage is described in the literature as a common morphological marker for necrotic chondrocytes [16, 17, 46] and was assumed in this study to be sufficient to recognize necrotic chondrocytes. A focal area in the epiphyseal growth cartilage containing cells with reduced size compared to the cell size in the surrounding cartilage was defined as a lesion with chondrocyte necrosis using the TPEF-signal. This was validated by comparison with an H&E -stained sample of an adjacent section.

#### Interpretation of SHG signals

Collagen fibrils within and surrounding the cartilage canals were characterized by the intensity of the SHG signal. The SHG signal arises from coherent amplification of the second harmonic waves emitted by the harmonophores, which were shown to be peptide bonds in the collagen triple helix [47]. The coherent amplification depends on how the triple helices are arranged with respect to each other within a specific length, known as the coherence length [41]. Typically, the intensity of the SHG signal increases linearly when the triple helices are randomly oriented and quadratically when they are aligned in the same direction [42]. Thicker collagen fibrils and fibrils ordered into bundles and fibres therefore give higher intensity [43, 48]. Most of the collagen in hyaline cartilage is of type II and is organized in a quasi-random network that give a speckle-like pattern in the SHG image [49]. In contrast, collagen fibrils that are highly ordered and oriented in the same direction at larger length scales, such as collagen type I fibres and fibril bundles, were recognized by several neighbouring high intensity pixels [38, 50].

#### Counting of cartilage canal segments

The study aimed to identify variations between different cartilage canals. To describe these variations quantitatively, the cartilage canals were divided into segments characterized by similar features as identified by the TPEF and SHG signals. A single cartilage canal located within a 100 μm-section represented a cross-section of a canal, and this cross-section could consist of one or more segments. Each segment of all the patent cartilage canals located within a 100 μm-section was then given an individual number in order to count the total number of patent cartilage canal segments. The percentage of segments with similar features was calculated.

## Results

### Variations between patent cartilage canals that may predispose to vascular failure

**Fig. 2:**
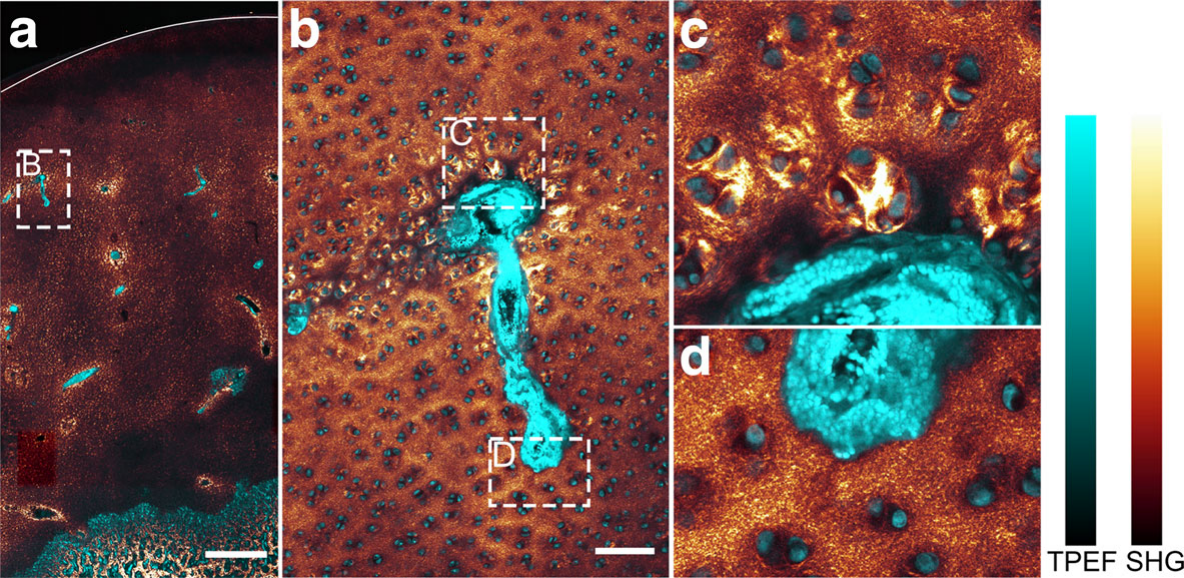
Variable collagen matrix surrounding cartilage canals. Cyan: Two-photon-excited fluorescence (TPEF), Glowing red: Second harmonic generation (SHG). **a** Section from the medial femoral condyle of the 82 day old pig. The cartilage canals are recognized by intense TPEF signal from their blood cells and perivascular cells and surrounded by variable SHG signal. The articular surface of the cartilage is indicated by a curved line. The layer beneath this curve with absence of cartilage canals is assumed to represent the immature articular cartilage. **b**-**d** Cross-section of a cartilage canal near its distal terminus (from the dashed rectangle in **a**). The end of the canal is magnified in **d** and primarily contains capillaries with densely packed erythrocytes giving intense TPEF signal. There is no obvious difference in the SHG signal between the matrix adjacent to and far from the cartilage canal. On the other hand, more proximal to the arterial source, adjacent cell groups are associated with intense SHG, while the SHG signal between the groups is low (magnified in **c**). This cross-section is therefore considered to consist of two different segments, one with no change in SHG signal of the cartilage matrix as a function of distance from the canal, and one that is surrounded by cells associated with intense SHG. 10×–focusing objective was used in **a**, and 25×–objective was used in **b**-**d**. The bar in **a** corresponds to 1 mm, while the bar in **b** equals 100 μm

The cartilage canals were present throughout the epiphyseal growth cartilage but were absent from the overlying immature articular cartilage (Fig. 2a). Patent cartilage canals were primarily observed in the 82-, 96- and 127-day old pigs, and only the patent canals in these pigs were counted. The TPEF and SHG signals revealed several variable features that were used to differentiate between segments of the patent cartilage canals.

#### Variation in the cell morphology and the collagen organization

In the cartilage matrix distant from a cartilage canal, the SHG intensity pixels formed a speckle-like pattern, signifying randomly oriented collagen fibrils. The same pattern was also observed immediately adjacent to some cartilage canals (e.g., in Fig. 2b). Cartilage canal segments where there was no obvious difference in the collagen matrix as a function of distance from the canal represented 41% (167/412) of the total number of segments (Table 1). These segments primarily contained capillaries filled with erythrocytes and were located towards the distal terminus of the branches.

**Table 1:** Fraction of patent cartilage canal segments characterized by different features

Pig age	82-day old	96-day old	127-day old	All three
(1) No change/total number of segments (%)	87/210 (41)	46/132 (35)	34/70 (49)	167/412 (41)
(2) Surrounded by intense SHG/total number of segments (%)	123/210 (59)	86/132 (65)	36/70 (51)	245/412 (59)
(3) Presence of collagen fibres/total number of segments (%)	59/210 (28)	36/132 (27)	17/70 (24)	112/412 (27)
(4) Indication of calcification/total number of segments (%)	22/210 (10)	8/132 (6)	0/70	30/412 (7)

Fraction of segments that (1) showed no change in SHG signal of the adjacent cartilage matrix as a function of distance from the canal, (2) was surrounded by cells associated with intense SHG signal, (3) had presence of collagen fibres at the canal margin, (4) was surrounded by high TPEF signal in the surrounding cartilage matrix as an indication of calcification

**Fig. 3:**
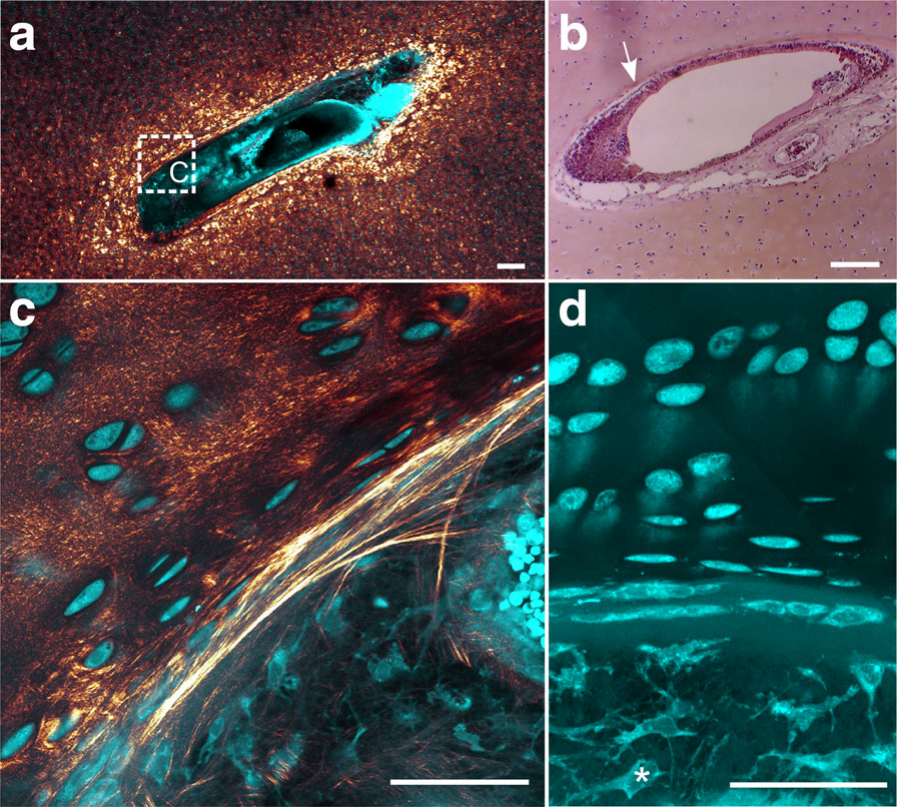
Cartilage canal with a boundary of collagen fibres. Cyan: Two-photon-excited fluorescence (TPEF), Glowing red: Second harmonic generation (SHG). **a** Cross-section of a cartilage canal surrounded by foci of intense SHG. **b** Hematoxylin and eosin-stained sample showing the same canal as in (**a**) but from an adjacent section. The canal has a margin that stains intensely eosinophilic (*arrow*). **c** A part of the canal (region indicated with the dashed rectangle in (**a**)) at higher magnification. Strands of high SHG intensity, indicating collagen fibres, appear to originate in the interior of the canal and align at the canal margin. **d** The TPEF signal gives the morphology of the various cell types at the both sides of the boundary between the cartilage canal and the adjacent cartilage. The interior of the canal contains cells with long processes (*asterisk*). At the boundary, the cell shape becomes flat, and in the adjacent cartilage, the cells are gradually more spherical. 10×–focusing objective was used in **a** and **b**, and 63× was used in **c** and **d**. All bars equal 100 μm

When travelling proximally along the cartilage canal from the distal terminus towards the arterial source, both the content of the canal and the SHG signal of the surrounding cartilage changed (compare Fig. 2c with 2D). The interior of the canal became more complex and consisted of an arteriole, one or more venules, varying number of capillaries and amount of perivascular tissue. Cells with long processes, presumed to represent fibroblasts, were occasionally present (Fig. 3). These were associated with strands of high SHG intensity, interpreted as collagen fibres (Fig. 3c). Some of the fibres extended outwards in an arcade-like structure and aligned at the cartilage canal margin to form a fibrous boundary. However, the fibrous boundary rarely encompassed the entire canal circumference. Comparison with an adjacent H&E-stained section containing the same cartilage canal showed that the fibrous boundary coincided with a margin that stained intensely eosinophilic (Fig. 3b). The cartilage canal segments characterized by the presence of collagen fibres constituted 27% (112/412) of the total number of segments (Table 1) and 48% (112/245) of the segments that were not located near the distal terminus.

In the adjacent cartilage, the cell morphology changed gradually from flat at the margin to almost spherical and chondrocyte-like more distant from the canal (Fig. 3d). The cells located within 200–500 μm from the canal margin were typically associated with intense SHG signal (e.g., in Fig. 2c and 3a). This signal probably reflects bundles of collagen fibrils oriented in the same direction around cells and cell groups. On the other hand, the SHG intensity from the matrix located between the cell groups was relatively weak in comparison. This pattern was observed in 59% (245/412) of the total number of patent cartilage canal segments.

#### Variation in the indication of calcification

**Fig. 4:**
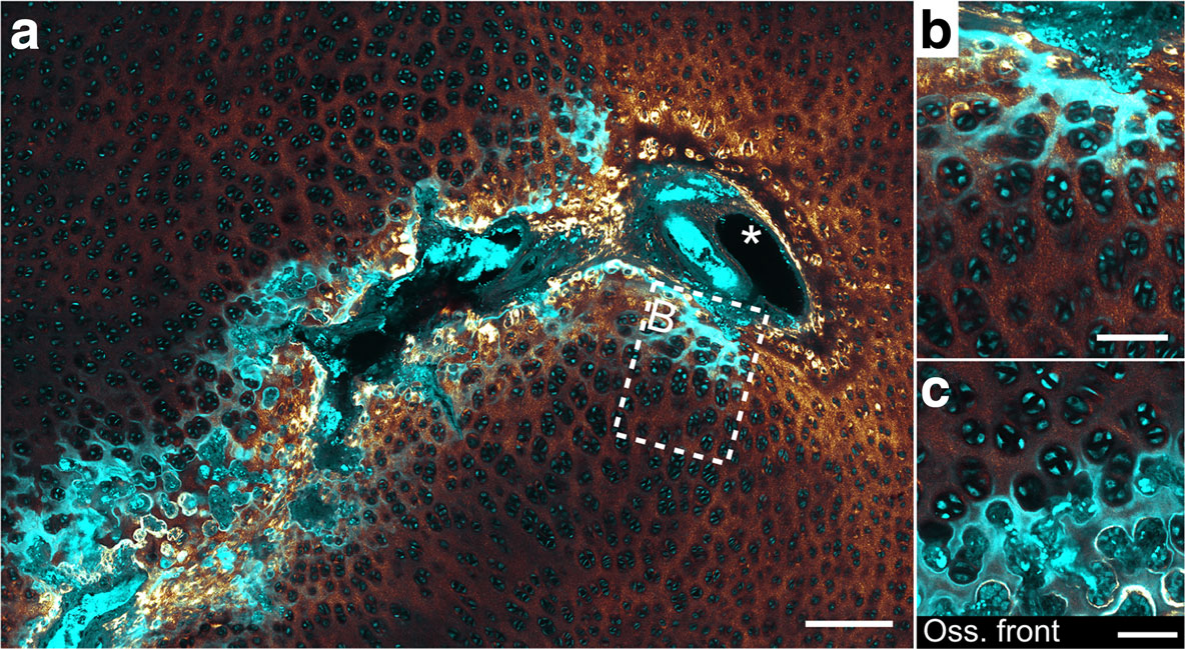
Indication of calcification around a cartilage canal. Cyan: Two-photon-excited fluorescence (TPEF). Glowing red: Second harmonic generation (SHG). **a** Cross section of a cartilage canal with variable presence of intense TPEF signal in the adjacent cartilage matrix. The intense TPEF signal is only located around the canal segment surrounded by hypertrophic chondrocytes, and the segment running into the image plane (asterisk) has no intense TPEF adjacent to it. **b** Close-up image of the dashed rectangle in **a**. **c** The intense TPEF of the calcified zone near the ossification front is shown for comparison. The TPEF signal is therefore presumably an indication of calcification. 25×–focusing objective was used to generate all images. The bars equal 250 μm in **a** and 100 μm in **b** and **c**

A strong TPEF signal was observed in the cartilage matrix surrounding some deep-located portions of cartilage canals that were surrounded by hypertrophic chondrocytes (Fig. 4). Segments characterized by this TPEF signal represented 7% (30/412) of the total number of segments (Table 1). The TPEF signal was comparable to the signal from the matrix of the presumed calcified zone near the ossification front (Fig. 4b compared to 4C) and was therefore interpreted as an indicator of calcification also around the cartilage canals.

#### Variation between patent cartilage canals crossing the osteochondral junction

**Fig. 5:**
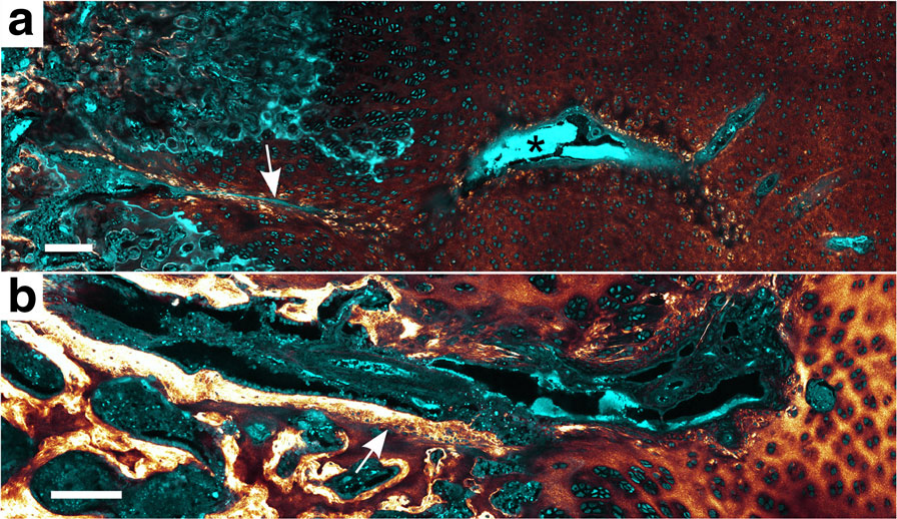
Patent cartilage canals crossing the osteochondral junction. Cyan: Two-photon-excited fluorescence (TPEF), Glowing red: Second harmonic generation (SHG). **a** A cartilage canal with a segment having intact blood vessels (*asterisk*) is narrowed and reduced to a streak at the ossification front (*arrow*). The streak was present in the entire 100 μm-section, indicating it was not an artefact. The canal is partly located outside the image plane. The narrowed segment is surrounded by intense SHG signal, clusters of hypertrophic cells, and more distant, a matrix of high TPEF signal indicating calcification. **b** A cartilage canal surrounded by layers of intense SHG signal, interpreted as collagen fibres or osteoid (arrow). The canal is not narrowed, and its lumen is connected with the bone marrow. 25×–focusing objective was used in both images. Bars equal 250 μm

The process of incorporating cartilage canals into the advancing SOC had different morphological effects on cartilage canals. The lumen of a cartilage canal was reduced to a streak at the ossification front (Fig. 5a). The streak was present in the entire 100 μm-section, which implied that it was not an artefact and that the continuity of the canal was interrupted at the osteochondral junction. The interruption had not affected the blood vessels located superficially in the canal, indicating that the interruption had occurred recently or that the vessels were still receiving blood from a canal located out-of-plane. Cartilage canals that were intact and not narrowed at the ossification front were surrounded by layers of intense SHG that probably represented osteoid (e.g., in Fig. 5b). The lumen of these canals was connected with the bone marrow.

### Location of sites where the blood supply had ceased

#### Chondrifying cartilage canals

**Fig. 6:**
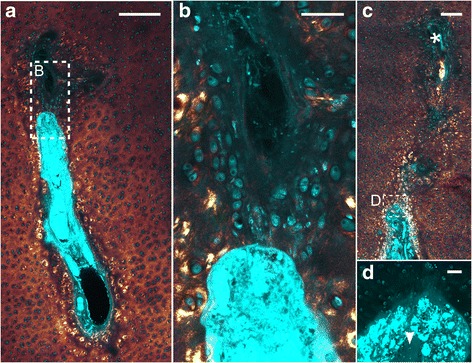
Chondrifying cartilage canals. Cyan: Two-photon-excited fluorescence (TPEF), Glowing red: Second harmonic generation (SHG). **a** The cartilage canal is chondrifying from its distal terminus. The chondrifying segment consists of degenerated blood vessels, debris and chondrocyte-like cells. More proximally, the canal has capillaries filled with blood cells giving intense TPEF. The SHG signal of the adjacent cartilage matrix is stronger around the segment contianing capillaries. **b** Higher magnification of the transition between the two segments of the canal. **c** A different chondrifying canal. The chondrifying segment (asterisk) is partly converted to cartilage and is several millimeters in length. Foci with intense SHG signal are located adjacent to the entire canal, but their number decreases more distally. **d** Higher magnification of the transition to the segment containing capillaries (only TPEF shown). Occasional chondrocyte-like cells are observed in this segment of the canal (arrow head). 25×–focusing objective was used to produce all images. The bars equal 250 μm in **a** and **c** and 50 μm in **b** and **d**

Most of the cartilage canals that were without intact blood vessels were chondrifying, and chondrifying cartilage canals were observed in (33/42) examined sections. Chondrifying cartilage canals were not associated with chondronecrosis in the adjacent cartilage. The process of replacing vascular tissue with cartilage had progressed furthest near the distal terminus (Fig. 6a). At this stage in the chondrification process, the canal was filled with chondrocyte-like cells and contained various fragments of high TPEF signal, interpreted as remnants of the vascular- and perivascular tissue (Fig. 6b, c). More proximally to the arterial source, there was an abrupt transition to vessels containing blood cells. However, chondrocyte-like cells were occasionally observed between the blood vessels in the canal (e.g., in Fig. 6d), and some of the vessels appeared degenerated. The site where the blood supply had ceased and the chondrification process initiated was presumed to be near this abrupt transition.

#### Necrotic cartilage canals were followed to the SOC or to the ligament attachment site

**Fig. 7:**
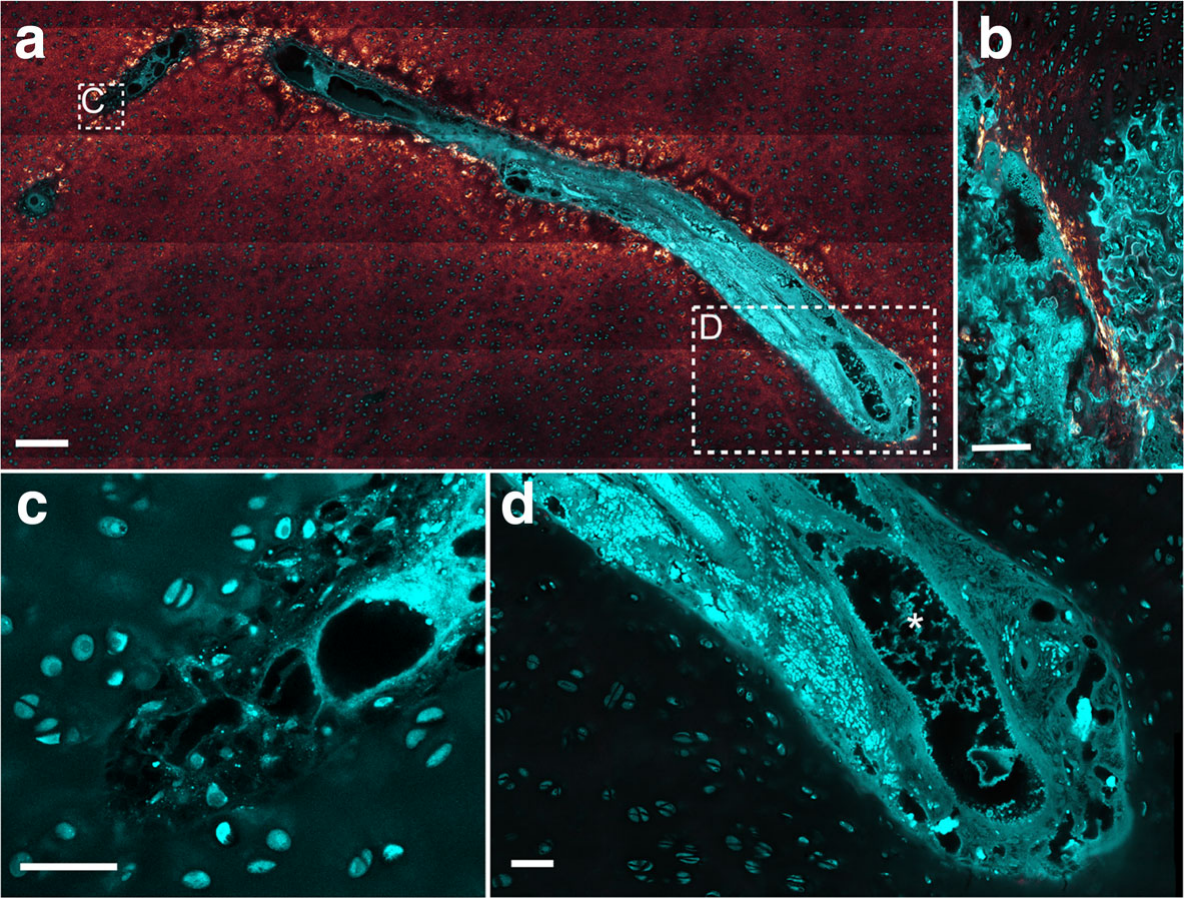
Necrotic cartilage canal. Cyan: Two-photon-excited fluorescence (TPEF), Glowing red: Second harmonic generation (SHG). **a** Cross-section of a cartilage canal containing degenerated blood vessels but with absence of chondrocytes in its lumen. The canal is therefore considered to be necrotic and not chondrifying. **b** At the site where the canal crosses the osteochondral junction, the canal appears to be disturbed by the ossification front, and a cone of cartilage persists around the canal into the ossification front. **c**-**d** Higher magnification of the regions indicated by dashed rectangles (only TPEF shown). Near the distal terminus, the canal contains remnants of blood vessels and fragments giving high TPEF signal, interpreted as debris or necrotic perivascular cells. More proximally to the arterial source, the canal is thicker and contains more complex tissue. However, the blood vessels and the perivascular tissue appear degenerated, and aggregating cells are present in the lumen of a vessel remnant (*asterisk*). The images were generated by a 25×–focusing objective. The bars correspond to 500 μm in **a** and **b** and are equal to 50 μm in **c** and **d**

**Fig. 8:**
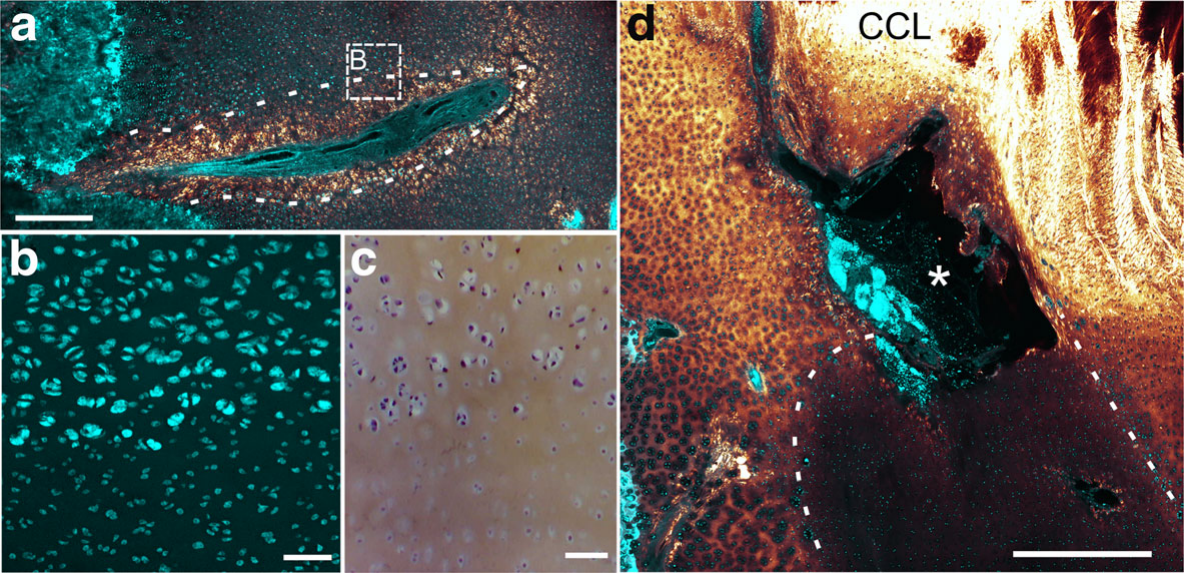
Necrotic cartilage canals associated with necrotic chondrocytes. Cyan: Two-photon-excited fluorescence (TPEF), Glowing red: Second harmonic generation (SHG). **a** An area with necrotic cells (indicated by dashed lines) is associated with a necrotic cartilage canal that enters the epiphyseal growth cartilage from the secondary ossification centre. The canal is surrounded by foci of intense SHG signal, associated with both necrotic and viable chondrocytes. Near the ossification front, the canal is narrowed. **b** The magnified TPEF-image shows the difference in cell size between the necrotic and viable chondrocytes. **c** A corresponding H&E-stained sample of an adjacent section verifies that the cell morphology can be visualized using TPEF. Some lacunae appear empty due to cell loss during sectioning. **d** A necrotic and dilated cartilage canal (asterisk) is associated with an area of necrotic chondrocytes (indicated by dashed lines). The canal originates from the caudal cruciate ligament (CCL) attachment site. The collagen fibres of the ligament give intense SHG signal. Accumulation of erythrocytes is observed within the necrotic canal. 10 x-focusing objective was used in **a**, while a 25×–objective was used in **b**. The bars are equal to 500 μm in **a** and **d** and 50 μm in **b** and **c**

**Fig. 9:**
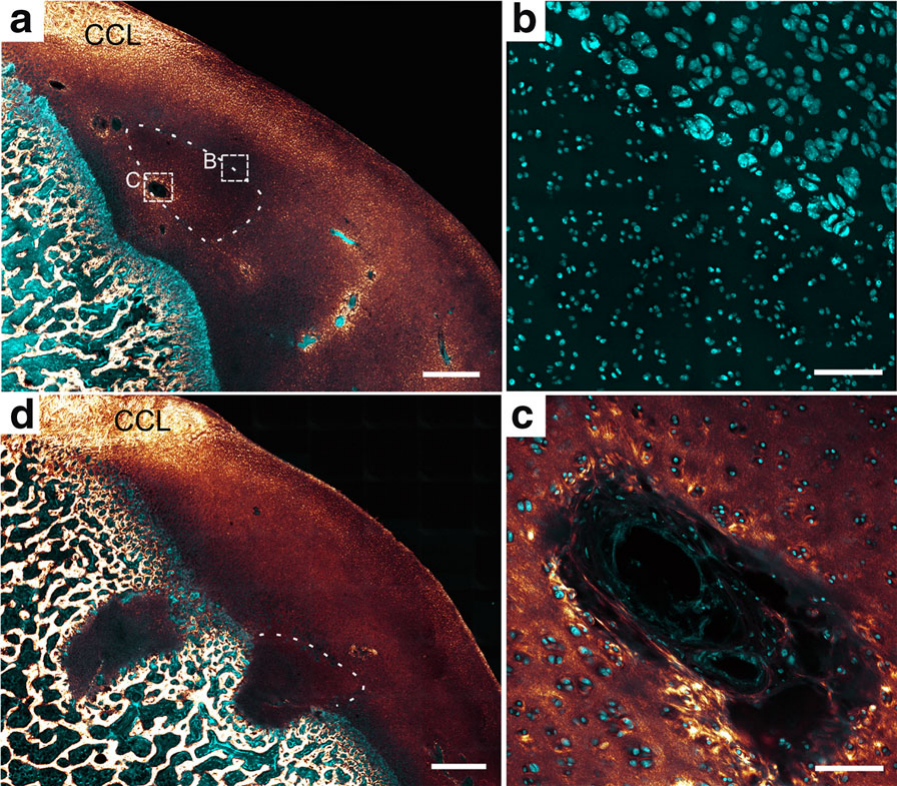
Necrotic cartilage and delay in ossification. Cyan: Two-photon-excited fluorescence (TPEF), Glowing red: Second harmonic generation (SHG). **a** An area with chondronecrosis is indicated by the dashed lines in the epiphyseal growth cartilage of the 82-day old pig. The chondronecrosis is located approximately 1.6 mm from the articular surface. The intense SHG signals above the lesion represent collagen fibres of the caudal cruciate ligament (CCL). **b** Magnified image of the dashed square that shows the border between the necrotic and viable cartilage. The necrotic chondrocytes appear shrunken. **c** Magnified image of the dashed rectangle showing a necrotic cartilage canal associated with the chondronecrosis. The chondrocytes located near the ossification front appear viable. **d** In the 140-day old pig, the areas with chondronecrosis have been surrounded by bone and have delayed the ossification. Images **a** and **d** were produced using a 10×–focusing objective, while a 25×–focusing objective was used in **b** and **c**. The bars equal to 1 mm in **a** and **d** and 100 μm in **b** and **c**

Necrotic cartilage canals were distinguished from chondrifying canals by their absence of viable chondrocytes in the canal lumen. The necrotic canals contained remnants of degenerated blood vessels and debris and were observed in all pigs and in 23/42 examined sections. Examples of necrotic canals are shown in Fig. 7-9. Most of the necrotic cartilage canals were surrounded by necrotic chondrocytes that were revealed by their smaller size compared to the adjacent non-necrotic chondrocytes (Fig. 8). However, the chondrocytes were not necrotic along the entire canal (e.g., Fig. 8d). 14 different necrotic cartilage canals could be followed until they crossed a boundary to the epiphyseal growth cartilage. This corresponded to the osteochondral junction in 11/14 cases and to the attachment site of the caudal cruciate ligament (CCL) in 3/14 cases. Necrotic cartilage canals associated with the CCL were not observed to cross the osteochondral junction. A transition to a patent segment was not found.

Necrotic cartilage canals connected to the SOC were oriented perpendicular to the ossification front and therefore likely represented vascular branches (e.g., in Fig. 7b, 8a). At the ossification front, their lumina were reduced in width, and it was not possible to follow them further into the SOC.

Necrotic cartilage canals coming directly from the ligament attachment site represented vascular trunks, and their lumina could be dilated and contain accumulation of blood cells (Fig. 8b).

Chondronecrosis was never located closer than 1.6 mm from the articular surface in any of the examined sample (e.g., in Fig. 9a), and segments of the necrotic cartilage canals located closer to the surface were chondrifying. In the 140-day old pig, some of the necrotic areas had caused delay in the ossification and were surrounded by bone (Fig. 9d).

Similar to the patent cartilage canals, the necrotic canals were surrounded by variable SHG signals as a function of distance from the canal, and a boundary of collagen fibres and adjacent foci of intense SHG were frequently observed (Fig. 7a, 8a, 9c).

## Discussion

In the current study, the possibility of using non-linear optical microscopy to reveal the cause of vascular failure was examined. The endogenous contrast provided by the SHG and TPEF signals was able to visualize cartilage canals in 100 μm-thick sections of the epiphyseal growth cartilage at a resolution comparable to conventional histology. Like other microscopic techniques, non-linear optical microscopy is destructive and requires appropriate sampling of animals. However, potential artefacts from sample preparation are reduced to a minimum due to the ability to image thick sections without staining.

The hypothesis that the presence of collagen fibres varied between segments of cartilage canals was supported. In the examined pigs, 27% of the patent cartilage canal segments were surrounded by a boundary of intense SHG strands that were interpreted as collagen type I fibres. These fibres were associated with fibroblast-like cells. Collagen type I has been demonstrated in cartilage canals of several species, including chicken [51], mouse [52], sheep [53], horse [32], and human [28, 54]. To the authors’ knowledge, this is the first study to indicate that such fibres also exist in porcine cartilage canals. The location of the fibres stained intensely eosinophilic in the current study. A comparable eosinophilic ring was observed around 69% of the cartilage canal cross-sections in the equine epiphyseal growth cartilage and stained for collagen type I after immunostaining [32]. The discrepancy between how much of the canals that was surrounded by these collagen fibres is probably caused by species- or maturation variance, and SHG and eosin may have different sensitivity to the collagen fibres. In addition, a single cartilage canal located in the 100 μm-section of the current study could consist of more than one segment, which could allow for more segments without collagen fibres to be included than when only considering cross-sections.

Collagen has long been proposed as a causative factor in the aetiology of OC [55, 10], but it has remained unclear how a primary disease in collagen would cause vascular failure in a single cartilage canal and leave most neighbouring canals intact [14]. Hellings et al. suggested that cartilage canals lacking a ring of collagen type I would be more vulnerable to vascular failure when they are incorporated into the SOC [32]. That cartilage canals that were intact and not narrowed at the osteochondral junction were surrounded by thick layers of collagen fibres supports this hypothesis. Being able to produce collagen type I fibres appears to be vital to avoid narrowing of canal lumen and potential vascular failure at the osteochondral junction. Canal segments lacking this ability may also lack the necessary cells or stimuli.

A potential cause of the narrowing of the lumen at the ossification front can be implied from the intense SHG signal associated with adjacent chondrocytes. This signal was interpreted to reflect bundles of collagen type II fibrils aligned in the same direction directly around the cells in contrast to a matrix of randomly oriented collagen fibrils between the cells. Such a pattern was suggested to be related to the chondrogenesis and cartilage matrix production known to occur around cartilage canals [38]. Perivascular mesenchymal cells in the canals can differentiate to chondrocytes that contribute to the cartilage growth [25]. In addition, a similar pattern of SHG signals was observed near the articular surface and the perichondrium where chondrogenesis also is known to occur [38]. Thus, production of cartilage matrix, cellular proliferation or hypertrophy around a cartilage canal at a site where there is no space to expand, e.g., at the ossification front, could potentially induce compression forces on the canal and subsequent narrowing, as hypothesized by Hurrel in 1934 [56]. However, due to the cross-sectional nature of the current study, it was not possible to determine if a narrow canal would cause vascular failure or if the reduced canal diameter was an effect of an already interrupted blood flow.

There was a strong TPEF signal in the cartilage matrix surrounding some of the cartilage canals located in the hypertrophic zone of the epiphyseal cartilage. The origin of the fluorescence is unknown, but when compared with the TPEF signal of the calcified zone near the SOC, it was interpreted to reflect calcified cartilage matrix. Because the samples were decalcified, the TPEF signal did not arise from the minerals per se, but probably fluorescent molecules or debris that were entrapped by the minerals. A comparable TPEF signal was also observed in the calcified zone of mature equine articular cartilage [35]. Calcification around cartilage canals occur during formation of the SOC, and after the SOC has been established, separate ossification foci can form around cartilage canals and later merge with the SOC [57]. Calcified matrix has been observed around cartilage canals in the human thyroid cartilage [54] and as a hyper-attenuating tube around cartilage canals with micro-CT [31]. Being surrounded by calcified tissue, in addition to collagen type I, before being incorporated into the SOC may be an advantage for the cartilage canal and prevent vascular failure. Both the role of calcification and collagen type I in the cause of vascular failure should be examined further.

Chondrification was shown to be an important cause of interrupted blood supply in a cartilage canal and appeared to be initiated near the distal terminus of a branch, in agreement with previous studies using histological observations [28, 17]. In contrast to vascular failure, chondrification is considered a physiological process that occurs in predictable patterns [22] and was not associated with chondronecrosis in this or previous studies [17, 18]. The perivascular cells and adjacent chondrocytes may therefore have adapted to a lower oxygen level when the blood supply ceases. Vascular regression is also found elsewhere, such as in the lens of the developing eye. The vascular system called tunica vasculosa lentis was assumed to regress because of blood flow constriction by smooth muscle contractions in the arterioles [58, 59]. Likewise, the same mechanisms may occur in the cartilage canals, possibly due to loss of survival factors (e.g., vascular endothelial growth factor) by the maturing cartilage [17]. Chondrification can be induced accidentally by surgically interrupting the blood flow in a cartilage canal [18–20, 60]. Portions of the transected cartilage canal located in the intermediate depth of the epiphyseal cartilage became surrounded by necrotic chondrocytes, while the superficial portions of the cartilage canal chondrified. Similarly, necrotic cartilage canals in the current study were not surrounded by necrotic chondrocytes when alternative sources of nutrition were nearby, e.g., near the articular surface, and these segments chondrified. The mechanisms that cause vascular failure can therefore also cause chondrification. It remains to be shown if the opposite also is true, i.e., if the mechanisms that cause chondrification also can cause vascular failure and ischemic chondronecrosis if they take place prematurely.

In the current study, it was possible to follow necrotic cartilage canals through optical and serial sections. In this way, 11 different canals were followed back to the SOC, and 3 canals originated from the attachment site of the cruciate ligament without crossing the osteochondral junction. The hypothesis that the blood supply ceases due to incorporation can therefore not be true in all cases. Nevertheless, incorporation was indicated to be the most common cause. That all necrotic cartilage canals connected to the SOC had their lumina narrowed to streaks at the ossification front supports the idea that vascular failure can occur during the incorporation process [14], potentially with a mechanism involving collagen type I or calcified matrix, as suggested above. However, if the narrowing is a consequence rather than a cause of the blood flow cessation, the vascular failure may have occurred at a deeper location, and evidence of the incidence would be removed by the advancing ossification front. In fact, CT-studies of pigs showed that 80% of the lesions were multi-lobular, i.e., occurring simultaneously and adjacent to each other [44]. A likely explanation for this is that the vascular failure normally happens in the vascular trunk and thus affects several branches at the same time [30, 44]. The necrotic cartilage canals connected to the ossification front in the present study were probably branches, and the location of the vascular failure was therefore not necessarily represented in the examined samples. To increase the likelihood of sampling an animal at the time of vascular failure, future studies should monitor the blood flow in the cartilage canals using in vivo methods, such as phase-contrast MRI if the spatial resolution can be improved [61]. Recent studies showed that cartilage canals could be visualized using susceptibly-weighted MRI [62, 36]. The contrast was assumed to be given by erythrocytes in the canal [62]. After surgically transecting blood vessels to the epiphyseal cartilage, cartilage canal abnormalities were only detected after three weeks [36]. This delay can be explained by the observations in the current study of erythrocytes in necrotic cartilage canals (e.g., Fig. 7).

That vascular failure also can occur at the attachment site of the caudal cruciate ligament has support in the literature Cartilage canals are able to enter the epiphyseal cartilage via this ligament [63], and the cause of vascular failure in these canals may be different from those that entered from the perichondrium and were incorporated into the SOC. Stretching forces from the ligament can possibly have ruptured blood vessels in these canals. Lesions of osteochondrosis have previously been associated with the attachment site of the cruciate ligament in pigs [17] and the short distal sesamoidean ligament in horses [64, 65]. In addition, lesions of osteochondritic dissecans in humans are known to commonly be located near the attachment site of the posterior cruciate ligament [66], which corresponds to the caudal cruciate ligament in pigs. Consequently, the cruciate ligaments can play a role in the aetiology of at least some cases of osteochondrosis, and vascular failure can therefore have different causes in different anatomical sites and regions. The ultimate goal is to determine the genes that predispose to osteochondrosis [14], and the results of the current study stress the importance of being aware that vascular failure can have different causes with different genetic predisposition. On the other hand, cartilage canals may be vulnerable to failure when crossing junctions between different tissues; bone-cartilage, ligament-cartilage, perichondrium-cartilage, and the genetic predisposition can therefore still be similar independent on location.

## Conclusion

The current study showed that non-linear optical microscopy can be used to image cartilage canals and early lesions of osteochondrosis in thick sections without staining. The hypothesis that cartilage canals of young pigs vary in their presence of collagen fibres at the canal margin was supported. In addition, a variation in the collagen fibril organization and calcification of the surrounding cartilage matrix was identified. The variable presence of these features gave a possible explanation for why only a subset of cartilage canals fails when they become incorporated into the SOC. The majority of the necrotic cartilage canals originated from the SOC and supported the prevailing hypothesis that vascular failure occurs during incorporation. However, 3/14 necrotic cartilage canals entered the epiphyseal growth cartilage from the attachment site of the caudal cruciate ligament without crossing the osteochondral junction. The role of this ligament in the cause of vascular failure and osteochondrosis should therefore be investigated in future studies.

## Abbreviations

SHG: Second harmonic generation
SOC: Secondary ossification centre
TPEF: Two-photon-excited fluorescence
